# Association between physical activity and health-related quality of life among adults in China: the moderating role of age

**DOI:** 10.3389/fpubh.2024.1334081

**Published:** 2024-03-27

**Authors:** Hongying Hao, Yemin Yuan, Jie Li, Dan Zhao, Peilong Li, Jingjie Sun, Chengchao Zhou

**Affiliations:** ^1^Centre for Health Management and Policy, School of Public Health, Cheeloo College of Medicine, Shandong University, Jinan, China; ^2^NHC Key Lab of Health Economics and Policy Research, Shandong University, Jinan, China; ^3^Shandong First Medical University and Shandong Academy of Medical Sciences, Graduate School, Jinan, China; ^4^China Center for Health Development Studies, Peking University, Beijing, China; ^5^Statistics and Evaluation Department of Shandong Health Commission Medical Management Service Center, Jinan, China

**Keywords:** physical activity, health-related quality of life, EQ-5D-3L, adults, cross-sectional

## Abstract

**Objective:**

The aim of the study was to examine the association between physical activity (PA) and health-related quality of life (HRQOL) among adults and explore the role of age in the association between PA and HRQOL in Shandong, China.

**Methods:**

We investigated the relationship between PA and HRQOL and examined the moderated role of age in this association among adults with different age groups and physical activity levels. Data were obtained from the sixth China National Health Services Survey conducted in Shandong province in 2018. The multi-stage-stratified cluster random sampling method was used to selected respondents, with individuals aged 18 and above included in the present study. The tool of assessing HRQOL was the three-level EuroQol Five Dimensions Questionnaire (EQ-5D-3L).

**Results:**

The study found PA was significantly related to HRQOL (*P* < 0.05). The interaction analysis indicated that the relationship between PA and HRQOL was significantly different across young, middle-aged, and older adults (*P* < 0.05). Older adults with the sufficient PA (coefficient = 0.090, 95%CI: [0.081, 0.100]) and active PA (coefficient = 0.057, 95%CI: [0.043, 0.072]) had significantly higher HRQOL compared with young and middle-aged groups.

**Conclusion:**

PA was positively associated with HRQOL among the adults. Age played a moderate role between the association between PA and HRQOL. Guidelines for PA should be specifically tailored to adults of different age groups in order to enhance their HRQoL.

## Introduction

As life expectancy continuously increases, health-related quality of life (HRQOL) has become a public health priority and a key goal for health system. The HRQOL is a multidimensional concept to reflect subjective wellbeing and ability to perform daily activities in different domains (1), which encompasses physical, mental health, self-perceived, and social domains related to health (2) and changes over time (3). The HRQOL has emerged as a crucial measure of health and is widely used to assess the overall health status of a population (4). It is an essential self-reported health outcome and indicator of wellbeing. Identifying relevant factors associated with HRQOL is meaningful to improve HRQOL. Previous studies reported that HRQOL was related to many characteristics, such as physical activity (5), education (6), social-economic status (7), smoking (8, 9), and sleep (10). Of these, physical activity (PA) is an important factor of HRQOL.

Physical activity (PA) is a skeletal muscle body movement that results in energy expenditure (11). Existing studies have proved that regular PA is associated with improvement of HRQOL (5, 10, 12–14), and PA is one of the most prevalent modifiable risk factors for acquiring disease worldwide (15). Existing study also found that PA was related to various aspects of HRQOL. One randomized controlled trail study found that the function of PA significantly enhanced physical functioning and mental health of adults in 40–74 years (16). Another study reported better physical health of the individual with sufficient PA than the individuals without insufficient PA and without PA (12). The positive function of PA on HRQOL was also found to be correlated with PA level, with populations at higher PA level generally experiencing better HRQOL (13, 17). Higher PA level was associated with better perception of HRQOL in the older adult, apparently healthy adults, and individuals with different clinical conditions (14). However, this relationship is not always linear under certain circumstances (18). Additionally, some studies have raised questioning the association among PA and HRQOL. A study reported that PA was not found to be correlated with the pain/discomfort dimension of HRQOL among the women (19). Another study indicated that randomized controlled trails and cohort studies provided limited evidence regarding the relationship between PA and HRQOL (17). Disparate findings among different studies regarding PA and HRQOL suggest the needs for further research in this area.

Age was found to be related to HRQOL (20, 21). Many studies focus on the relationship between PA and HRQOL, but most have concentrated on specific age groups, such as children (3), adolescents (22), students (23), middle-age population, or older adult population (24–28). The conclusions drawn from these studies consistently indicated that higher PA levels are associated with better HRQOL (3, 23, 29). Previous research on HRQOL among older adult populations has shown that the oldest adults reported worse HRQOL than the younger older adult individuals (21, 30). However, the study on PA among old adults reported that PA can improve the HRQOL of adults (31). Additionally, this positive impact of PA on HRQOL is more pronounced in younger old than older adults, and being physically active earlier in life is beneficial for successful aging later on Lin et al. (32). The study findings indicate that the impact of physical activity (PA) on the health-related quality of life (HRQOL) of adults may vary across different age groups. It is speculated that age might act as a moderating factor in the relationship between PA and HRQOL. Moreover, to analyze this relationship while considering other factors, we controlled for covariates such as marital status, education level, smoking, alcohol consumption, and chronic diseases.

The general goal of this study was to investigate the relationship between PA and HRQOL in adults residing in Shandong province, China. The specific objectives are as follows: (1) to explore the association between PA and HRQOL, different PA levels, and HRQOL in adults and (2) to examine the potential moderating role of age on the association among PA and HRQOL. Clarifying the association between PA and HRQOL among different age groups will provide new evidence for policymakers to design general and age-specific health intervention programs at the national health level and to encourage adults to adopt healthy behaviors.

## Methods

### Data and design

This study used the data from the sixth China National Health Services Survey in Shandong province in 2018. Shandong is located in the eastern China, which is the second most populous province in China. The population exceeded 101.7 million in 2020, of which 66.1% were aged 15–64 years (33). The China National Health Services Survey was conducted by the Statistics Information Center of National Health Commission of the People's Republic of China since 1993. The follow-up survey was conducted every 5 years. The multi-stage-stratified cluster random sampling method was used (34). A total of 20 counties (districts) of 17 municipalities in Shandong Province were included. Each county (districts) randomly sampled five towns or streets. Two villages (communities) were sampled from each town (street). Households were extracted from villages (communities) by a system sampling method, and 65 households were selected from each village (community), with individuals aged 18 and above included in present study. The data were collected through face-to-face interview by trained local investigators using electronic data collection devices.

## Measures

### Health-related quality of life

In this study, the HRQOL was a dependent variable. The instrument used to evaluate the HRQOL was the three-level EuroQol Five Dimensions Questionnaire (EQ-5D-3L) (35), which has become an important scale for measuring HRQOL in the world (21, 36). The validity and reliability of EQ-5D-3L had examined among Chinese population (37–42). EQ-5D-3L includes five dimensions, namely, mobility, self-care, usual activities, pain/discomfort, and anxiety/depression. Each dimension contains three levels: no problems, some problems, and extreme problems. Because the percentage of extreme problems was low in each dimension, for echoing in previous study (43), we divided participants in each dimension into no problem (0) and have problem (including some problem and extreme problem, 1) (9). The EQ-5D-3L utility value was generated by the Chinese time trade-off method (44). The Chinese utility values for EQ-5D-3L health status ranged from −0.149 to 0.887 (44), which were similar to those estimated in other counties. The value of 0.887 represents full health and negative value represents health status worse than death, which indicated that the HRQOL of individuals was extremely poor (45).

### Physical activity

Physical activity (PA) was an independent variable in this study. International Physical Activity Questionnaire Short Version (IPAQ-SF) (46) was usually used to collect the information of PA in the past 7 days: the average time per day, the number of day physical activity per week, and the intensity of the PA. The China National Health Services Survey draw on the methods of IPAQ-SF collecting PA time and frequency, but the collection period was adjusted to the past 30 days per week. The questions were as follows: “*In the past 30 days, how many days per week did you engage in moderate or vigorous physical activities (such as morning exercises, inter-class exercises, physical education classes, extra-curricular sports classes, work-room exercises, square dancing, walking exercises, walking, running, etc.)?,” “How many minutes did you spend exercising each time?,” and “How intense is your average physical activity (self-breathing and heart rate increase).”*

Based on the recommended physical activity (PA) guideline of the World Health organization (WHO) (47) and American College of Sports Medicine (ACSM) Position Stand (48–50), along with previous study about physical PA levels and HRQOL (12, 18, 51), we chose to follow the minimum standard of recommended guideline for this study. Consequently, PA was classified into three levels:

1) inactive PA: no physical activity;

2) insufficient PA: participated in some physical activity but did not meet the recommend line;

3) active PA: moderate physical activity for at least 30 min·d on 5 d·wk or vigorous physical activity for at least ≥20 min·d on ≥3 d·wk.

### Covariates

The age group was divided into three age categories (51–53): the young group (18–44 years), middle-aged group (45–64 years), and older group (≥65 years). Marital status was divided into married and single (including divorced, unmarried, and widowed). The educational level was coded as four groups: illiteracy, primary and junior, senior, college and above. In addition, smoke, drink, and chronic diseases were included in this study (Table 1).

**Table 1:** Basic characteristics of participants by physical activity level in Shandong province, China.

**Characteristics**		**Frequency (%)**	**Physical activity level**			***P*-value**
			**Inactive (%)**	**Insufficient (%)**	**Active (%)**	
N (%)	27,673 (100.00)	13,978 (50.51)	3,220 (11.64)	10,475 (37.85)	
Age	Young adults	9,926 (35.87)	5,136 (51.74)	1,577 (15.89)	3,213 (32.37)	< 0.001
	Middle-aged adults	11,804 (42.66)	6,025 (51.04)	1,140 (9.66)	4,639 (39.3)	
	Older adults	5,943 (21.48)	2,817 (47.40)	503 (8.46)	2,623 (44.14)	
Gender	Female	14,407 (52.06)	7,150 (49.63)	1,713 (11.89)	5,544 (38.48)	0.009
	Male	13,266 (47.94)	6,828 (51.47)	1,507 (11.36)	4,931 (37.17)	
Marital status	Married	24,193 (87.42)	12,330 (50.97)	2,792 (11.54)	9,071 (37.49)	< 0.001
	Single	3,480 (12.58)	1,648 (47.36)	428 (12.3)	1,404 (40.34)	
Education	Illiteracy	3,597 (13.00)	2,170 (60.33)	308 (8.56)	1,119 (31.11)	< 0.001
	Primary and junior school	15,390 (55.61)	8,464 (55.00)	1,525 (9.91)	5,401 (35.09)	
	Senior school	4,906 (17.73)	2,119 (43.19)	643 (13.11)	2,144 (43.7)	
	College and above	3,780 (13.66)	1,225 (32.41)	744 (19.68)	1,811 (47.91)	
Tobacco	No	21,784 (78.72)	10,588 (48.6)	2,549 (11.7)	8,647 (39.69)	< 0.001
	Yes	5,889 (21.28)	3,390 (57.56)	671 (11.39)	1,828 (31.04)	
Alcohol	No	19,308 (69.77)	9,669 (50.08)	2,255 (11.68)	7,384 (38.24)	0.026
	Yes	8,365 (30.23)	4,309 (51.51)	965 (11.54)	3,091 (36.95)	
Chronic diseases	No	19,391 (70.07)	9,860 (50.85)	2,424 (12.5)	7,107 (36.65)	< 0.001
	Yes	8,282 (29.93)	4,118 (49.72)	796(9.61)	3,368 (40.67)	

### Statistical analysis

First, frequency and percentage were used to describe the demographic characteristics of the adults by different age groups. Second, the analysis of variance (ANOVA) was employed to compare the differences of participants' EQ-5D-3L index values among adults with different PA levels and age groups. Third, ordinary linear regression analysis was employed to investigate the relationship between PA (independent variable) and HRQOL (dependent variable). We first introduced PA and age into regression mode l. Then, we added the interaction of PA level and age into model 2. If the interaction term was significant, we would analyze the association among PA and HRQOL stratified by age. Stata 14.0 is used to analyze all data.

## Results

### Social-demographic characteristics

Table 1 presents the basic characteristics of respondents by the PA level. A total of 27,673 adults were included in the present study. Out of the total 27,673 participants, 50.51% were inactive, and 37.85% were active. The majority of the participants were middle-aged adults (42.66%), female (52.06%), primary and junior education (55.61%), without tobacco consumption (78.72%), without alcohol consumption (69.77%), and without chronic diseases (70.07%).

### The EQ-5D-3L utility values of adults by PA level and age

As shown in Table 2, the average EQ-5D-3L utility value was 0.889. Adults with active PA had the highest EQ-5D-3L utility values (0.907), and adults with inactive PA level had the lowest values (0.873). The results of ANOVA showed there was a significant difference (*P* < 0.001) in EQ-5D-3L utility values across different PA levels of general adults. The pairwise comparison results further showed a significant difference (*P* < 0.001) in EQ-5D-3L utility values of adults in different levels of PA. Adults in active PA level had the highest EQ-5D utility values in all age groups, and the adults with inactive PA level had the lowest EQ-5D utility values in all age groups. When stratified by age, there are significant differences (*P* < 0.001) in EQ-5D-3L utility values across PA levels among all age groups.

**Table 2:** EQ-5D-3L utility values by PA level and age group and the percentage of adults with problems in EQ-5D in Shandong province, China.

**Variable**	**EQ-5D-3L utility values, mean ±SD**	**The percentage of adults with problems in EQ-5D**				
		**MO (%)**	**SC (%)**	**UA (%)**	**PD (%)**	**AD (%)**
Total^***^ b^***^ (*n* = 27,673)	0.889 ± 0.001	9.29	4.29	6.59	19.20	6.45
Inactive	0.873 ± 0.001	12.28	6.27	9.13	22.63	8.30
Insufficient	0.900 ± 0.002	6.93	2.55	4.63	15.47	4.19
Active	0.907 ± 0.001	6.02	2.18	3.81	15.76	4.47
Young adults (*n* = 9,926) 1^***^		1.28	0.88	1.36	5.29	2.54
Inactive	0.923 ± 0.001	1.71	1.11	1.95	6.52	3.33
Insufficient	0.929 ± 0.001	1.01	0.76	1.01	4.57	1.65
Active	0.932 ± 0.001	0.72	0.56	0.59	3.67	1.71
Middle-aged adults (*n* = 11,804) 2^***^		7.95	2.91	5.12	21.72	7.06
Inactive	0.879 ± 0.002	10.42	4.00	6.90	25.56	8.85
Insufficient	0.898 ± 0.003	6.75	2.28	3.95	21.05	6.93
Active	0.908 ± 0.001	5.04	1.64	3.08	16.90	4.76
Older adults (*n* = 5,943) 3^***^		25.31	12.27	18.26	37.41	11.80
Inactive	0.770 ± 0.004	35.50	20.52	26.98	45.72	16.19
Insufficient	0.834 ± 0.007	25.84	8.75	17.50	36.98	10.54
Active	0.874 ± 0.002	14.26	5.11	9.04	28.56	7.32

MO, mobility; SC, self-care; UA, usual activity; PA, pain or discomfort; AD, anxiety or depression. *P*-values with statistical significance: ^***^P < 0.001. ^a^ANOVA tests was employed to compare the EQ-5D utility values among adults in PA levels. ^b^ANOVA test was used to compare the EQ-5D utility values between age groups and PA levels. ^1, 2, 3^ Bartlett's test was used to compare the EQ-5D utility values of adults with each PA level in young, middle-age, and older adults, respectively.

The percentage of adults with EQ-5D problem in inactive PA level (pain/discomfort: 22.63%, mobility: 12.28%, usual activity: 9.13%, anxiety/depression: 8.3%, and self-care: 6.27%) is higher than that of adults in insufficient (pain/discomfort: 15.47%, mobility: 6.93%, usual activity: 4.63%, anxiety/depression: 4.19%, and self-care: 2.55%), and active PA level (pain/discomfort: 15.76%, mobility: 6.02%, usual activity: 3.81%, anxiety/depression: 4.47%, and self-care: 2.18%). Older adults in each PA level tended to report more problems in EQ-5D. Adults with inactive PA level reported more problems in all EQ-5D-3L dimensions.

### Association between PA levels and HRQOL among adults

In Table 3, after adjusting for gender, marital status, education, tobacco consumption, alcohol consumption, and chronic diseases, model 1 showed that PA was positively association with HRQOL of adults. Compared with adults with inactive PA, adults with insufficient PA (coefficient = 0.019, 95%CI: [0.015, 0.023]) and active PA (coefficient = 0.035, 95%CI: [0.032, 0.038]) reported better HRQOL. When adding the interaction term of PA levels and age, model 2 showed that the moderating role of age in the association between PA levels and EQ-5D-3L utility values was still significant (*P* < 0.001).

**Table 3:** Relationship between physical activity and HRQOL among adults in Shandong province, China.

**Characteristic**	**Model 1: No interaction**		**Model 2: PA level** × **age**	
	**Coefficient**	**95%CI**	**Coefficient**	**95%CI**
**Physical activity level**				
Inactive	1.0		1.0	
Insufficient	0.019^***^	[0.015, 0.023]	0.003	[0.03, 0.038]
Active	0.035^***^	[0.032, 0.038]	0.007^***^	[0.011, 0.023]
**Age**				
Young adults	1.0		1.0	
Middle-aged adults	−0.032^***^	[−0.034, −0.029]	−0.038^***^	[−0.034, −0.029]
Older adults	−0.087^***^	[−0.092, −0.081]	−0.129^***^	[−0.092, −0.082]
**PA level** × **Age**				
Insufficient × middle-aged group			0.012^***^	[0.005, 0.019]
Insufficient × older group			0.059^***^	[0.043, 0.075]
Active × middle-aged group			0.018^***^	[0.013, 0.023]
Active × older group			0.088^***^	[0.078, 0.097]
*R^2^*	0.146		0.162	
*R^2^*change	0.114		0.113	
F	338.36^***^		255.650^***^	

Adjusted for gender, marital status, education, tobacco, alcohol, and chronic diseases. ^***^P < 0.001.

Table 4 shows the results stratified by age, the relationship of PA level, and each dimension of EQ-5D. After adjusting for gender, marital status, education, tobacco consumption, alcohol consumption, and chronic diseases, insufficient PA level and active PA level were significantly associated with HRQOL in middle-aged and older adults. For young adults, active PA level was significantly associated with HRQOL. Middle-aged adults with sufficient (coefficient = 0.016, 95%CI: [0.009, 0.022]) and active PA level (coefficient = 0.026, 95%CI: [0.022, 0.030]) reported higher EQ-5D-3L utility values. This positive association was also observed among old adults with insufficient (coefficient = 0.065, 95%CI: [0.050, 0.080]) and active PA (coefficient = 0.095, 95%CI: [0.086, 0.105]) level. Except the dimensions of pain/discomfort and anxiety/depression of middle-aged adults in insufficient PA level, the percentages of EQ-5D dimensions with problem were all associated with PA. Adults with insufficient PA reported more problems in dimensions except for anxiety/depression among young adults.

**Table 4:** Association between physical activity and HRQOL among young, middle-aged, and older adults in Shandong province, China.

**Characteristics**	**EQ-5D-3L values coefficient^a^ (95%CI)**	**EQ-5D-3L dimensions**				
		**MO AOR**^b^ **(95%CI)**	**SC AOR**^b^ **(95%CI)**	**UA AOR**^b^ **(95%CI)**	**PD AOR**^b^ **(95%CI)**	**AD AOR**^b^ **(95%CI)**
**Young adults (*****n*** = **9,926)**						
Inactive	Ref	1.0	1.0	1.0	1.0	1.0
Insufficient	0.003 [0.000, 0.005]	0.91 [0.526, 1.590]	1.07 [0.557, 2.059]	0.83 [0.474, 1.455]	0.87 [0.663, 1.130]	0.60^***^ [0.394, 0.922]
Active	0.005^***^ [−0.003, 0.008]	0.52^***^ [0.321, 0.848]	0.579 [0.326, 1.027]	0.34 [0.202, 0.582]	0.67^***^ [0.535, 0.831]	0.55^**^ [0.399, 0.756]
**Middle-aged adults (*****n*** = **11,804)**						
Inactive	Ref	1.0	1.0	1.0	1.0	1.0
Insufficient	0.016^***^ [0.009, 0.022]	0.68^***^ [0.523, 872]	0.57^***^ [0.373, 0.866]	0.58^***^ [0.419, 0.804]	0.84 [0.717, 0.985]	0.81 [0.442, 0.624]
Active	0.026^***^ [0.022, 0.030]	0.50^***^ [0.428, 0.593]	0.40^***^ [0.308, 0.529]	0.45^***^ [0.368, 0.553]	0.65^**^ [0.583, 0.914]	0.54^***^ [0.636, 1.011]
**Older adults (*****n*** = **5,943)**						
Inactive	Ref	1.0	1.0	1.0	1.0	1.0
Insufficient	0.065^***^ [0.050, 0.080]	0.60^***^ [0.483, 0.751]	0.34^***^ [0.248, 0.479]	0.54^***^ [0.415, 0.689]	0.68^***^ [0.445, 0.565]	0.60^***^ [0.445, 0.820]
Active	0.095^***^ [0.086, 0.105]	0.32^***^ [0.277, 0.367]	0.22^***^ [0.176, 0.264]	0.27^***^ (0.232, 0.322)	0.52^***^ [0.597, 0.849]	0.47^***^ [0.390, 0.563]

Adjusted for gender, marital status, education, tobacco, alcohol, and chronic diseases. MO, mobility; SC, self-care; UA, usual activity; PA, pain or discomfort; AD, anxiety or depression. *P*-values with statistical significance: ^***^P < 0.001. ^a^Linear regression analysis stratified by age group without covariable adjustment. ^b^Odds ratio based on logistic regression analysis fully adjustment. ^**^P < 0.05.

## Discussion

This study found that there was a positive association between PA and HRQOL of adults among young, middle-age, and older adults in Shandong province, China. The same activity level played different roles in HRQOL among different age groups. This finding helps elucidate that age is an important factor in the relationship between PA and HRQOL and facilitates individuals choose suitable type of PA so as to improve HRQOL.

Echoing previous study (16), our results showed that PA was positively associated with HRQOL among general adults in China. First, PA could improve cardiorespiratory fitness (commonly measured by maximal oxygen uptake), which is a most important indicator of health. PA could improve numerous factors that limited maximal oxygen uptake (54), and after aerobic training, the maximal oxygen uptake could increase (55) and the population would release stress, reduce anxiety, and improve physical health and mental health. Furthermore, the relationship between PA and HRQOL was clearer by the analysis by the PA level. In active PA level, we found that PA was positively associated with better HRQOL among general adults. These finding were consistent with previous studies that reported active participants had better HRQOL than inactive and insufficiently active participants (12, 14, 18, 56), but in insufficient PA level, this positive relationship among PA and HRQOL was not obvious among the young adults. Meanwhile, our finding provides further evidence of the relationship among PA dose and HRQOL. The function may be limited, but insufficient PA level is also positive to HRQOL of middle-age and older adults. These findings suggest that it is essential to do moderate PA to improve HRQOL for adults.

In the present study, we found that age was not only associated with HRQOL but also moderated the positive effect of PA on HRQOL. With the increase of age, HRQOL decreased, which was in accordance with previous study (57). The moderated analysis showed that the improvement of PA on HRQOL was most evident in older adults, indicating that age moderated the positive function of PA to HRQOL and the positive function was more conspicuous in older adults. This finding was in accordance with previous studies that strong evidence on PA improves HRQOL of older adults and limited evidence of young adults (20, 58). An interpretation for this finding might be that older adults have enough time to engage in PA regularly. Another reason might be that HRQOL of older adults decreased significantly with the increase of age; thus, the effect of PA on HRQOL in older adults may be more visible than middle-age and young adults. The findings indicated that future interventions should pay more attention to the older individuals.

We found most dimensions of HRQOL were significantly associated with PA, especially among middle-aged and older adults. For middle-aged adults, the function of PA on HRQOL was also obvious, except the dimension of pain or discomfort. This finding was consistent with the abovementioned research (59, 60). Similar finding was reported in previous study from different perspectives that PA-induced changes in neurotransmitters of the brain and endogenous opioids were associated with mental health, such as depression, anxiety, and other mood constructs (27, 61, 62). We also found that PA significantly improved all HRQOL dimensions of older adults. This indicated that older adults engaging PA could effectively enhance all dimensions of HRQOL. As for the young adults, the function of PA was significant in the dimension of anxiety or depression. This finding implied that policymaker should develop different PA interventions for the adults with different ages to enhance HRQOL.

This study has several limitations. Frist, the data used in present study are cross-sectional, which could not determine the causal relationship between PA and HRQOL. Second, HRQOL is self-reported, which might result in some potential bias. The PA level was divided by the minimum standard of recommend guideline, which may be overestimating the population with active PA. Third, this study is based on the data from Shandong province, and the main conclusion or findings may not be generalized to other provinces or regions.

## Conclusion

This study shown that PA was positively related to of HRQOL, and most of HRQOL dimensions were significantly associated with PA. The association between PA and HRQOL was found to be moderated by age, with the influence of PA on the HRQOL of older adults being more significant than that of other age groups. These findings offer new evidence for policymakers to develop targeted interventions aimed at effectively improving HRQOL for different age groups of adults.

## Data availability statement

The datasets presented in this article are not readily available because the Data Confidentiality Regulations of Shandong Provincial Medical Management Center. Requests to access the datasets should be directed to JS, sunjingjie163@163.com.

## Author contributions

HH: Data curation, Formal analysis, Software, Writing—original draft. YY: Data curation, Writing—review & editing. JL: Project administration, Resources, Supervision, Writing—review & editing. DZ: Writing—review & editing. PL: Writing—review & editing, Investigation, Project administration. JS: Investigation, Project administration, Resources, Writing—review & editing. CZ: Project administration, Conceptualization, Methodology, Supervision, Validation, Writing—review & editing.
